# Gender imbalance in intensive care: High time for action and evaluation!

**DOI:** 10.1186/s13054-021-03657-8

**Published:** 2021-07-07

**Authors:** Caroline Hauw-Berlemont, Charlotte Salmon Gandonnière, Florence Boissier, Nadia Aissaoui, Laetitia Bodet-Contentin, Muriel Sarah Fartoukh, Mercedes Jourdain, Julien Le Marec, Fabienne Tamion, Olfa Hamzaoui, Cécile Aubron, the FEMMIR (Femme Médecins en Médecine Intensive Réanimation) Group for the French Intensive Care Society, Caroline Hauw-Berlemont, Charlotte Salmon Gandonnière, Florence Boissier, Nadia Aissaoui, Laetitia Bodet-Contentin, Muriel Sarah Fartoukh, Mercedes Jourdain, Julien Le Marec, Fabienne Tamion, Olfa Hamzaoui, Cécile Aubron

**Affiliations:** 1 Médecine Intensive Réanimation, Hôpital Européen Georges Pompidou, AP-HP, Université de Paris, Paris, France; 2 Médecine Intensive Réanimation, INSERM CIC 1415, CRICS-TriGGERSep Network, CHRU de Tours, Tours, France; 3 Médecine Intensive Réanimation, Hôpital Universitaire de Poitiers, Poitiers, France; 4 INSERM CIC 1402 (ALIVE Group), Université de Poitiers, Poitiers, France; 5 INSERM Unit 970, Cardiovascular Research Center (PARCC), Hôpital Européen Georges Pompidou, APHP, Université de Paris, Paris, France; 6 Médecine Intensive Réanimation, INSERM CIC 1415, CRICS-TriGGERSep Network, CHRU de Tours and methodS in Patient-Centered Outcomes and Health ResEarch (SPHERE), INSERM UMR 1246, Université de Tours, Tours, France; 7 Service de Médecine Intensive Réanimation, Faculté de Médecine Sorbonne, Université, Hôpital Tenon, APHP, Sorbonne Université, Paris, France; 8 Médecine Intensive Et Réanimation - CHU de Lille, Lille, France; 9 Membre de L’unité INSERM U1190 - Recherche Translationnelle Sur Le Diabète, Lille, France; 10 Site Pitié-Salpêtrière Charles Foix, Service de Pneumologie, Médecine Intensive Réanimation, Département R3S, AP-HP Sorbonne Université, Paris, France; 11 INSERM, UMRS1158 Neurophysiologie Respiratoire Expérimentale Et Clinique, Sorbonne Université, Paris, France; 12 Médecine Intensive Réanimation, Hôpital Universitaire de Rouen, Rouen, France; 13 INSERM U1096 EnVi, Université Normandie, UNIROUEN, Rouen, France; 14 Service de Réanimation Polyvalente, Hôpital Antoine Béclère, AP-HP, Université Paris-Saclay, Clamart, France; 15 Médecine Intensive Réanimation, Centre Hospitalier Régional et Universitaire de Brest, Université de Bretagne Occidentale, Brest, France

There is an accumulating evidence of persistent gender inequity in medicine including intensive care. In Science, the recognition of gender imbalance and the call for actions to redress it, has been reported since some years ago [1]. Unfortunately, the situation seems to last: recent literature in medicine has shown that women underrepresentation in leadership positions has not improved over the last 35 years [2–4]. Recent studies reported high rates of gender discrimination towards women intensivist with an impact on their professional and personal development [4, 5].

Assessing gender inequity is a key element of the policies fighting against women vertical segregation. However, awareness of the situation and willingness to change might not be sufficient for a real shift in our behaviors and for allowing gender equity in intensive care in the near future.

Specific actions as those listed recently by Vincent et al. [6] to improve gender balance have been proposed many times in numerous contexts [7]; however many authors report the lack of qualitative evaluable measures to determine whether changes have been implemented and have led to better gender equity.

It is urgent for our intensive care community to actively implement measurable actions and metrics to reach better equity but also to evaluate the impact of those actions (Fig. 1). Words proved themselves insufficient. There will be no spontaneous transformation of what has been happening for ages and we want to warn on the urgent necessity to change the “standard of our behaviors.”

**Fig. 1:**
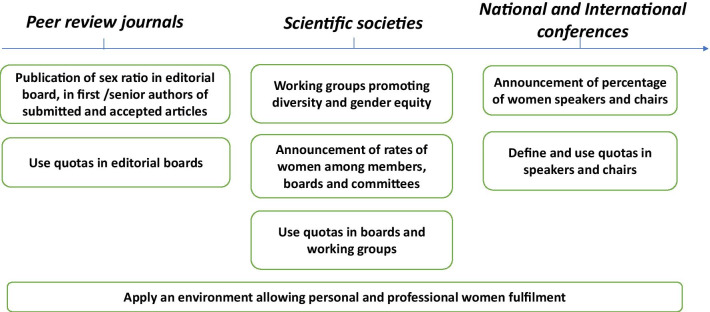
Example of concrete actions to reach awareness of gender imbalance and to achieve gender equity in intensive care

Working groups to guarantee diversity and equity are crucial to assure identification, implementation and respect of measures to reach better gender balance. The French Intensive Care Society already created the FEMMIR (FEmmes Médecins en Médecine Intensive Réanimation) group in 2019 to promote women in intensive care.

Vranas et al*.* showed that a profound gender gap in critical care literature still exists in 2020 [8]. What obstacles can possibly be argued not to take measures in critical care publications to reverse the current trend?

As an example, critical care journals could trigger positive moves and take the lead by proposing the implementation of different measures that proved themselves efficient [9]:

- Journals can use quotas to improve gender balance within the editorial boards. Although quotas remain debated, considered unfair and inequitable by some, it must be understood that they are essential to succeed in achieving gender balance. Quota must not be considered as an outcome but as a tool to reach equity. Once the parity will be reached and sustained, this temporary measure will end.
- All peer review journals should annually publish percentages of women members in their editorial board, of women who have submitted/published a paper as a first or senior author and of women who have reviewed a submitted manuscript. Raising awareness on those numbers can lead to substantial changes.
- National and international conferences should announce the percentage of women speakers and chairs on their program.
- National and international societies of Intensive Care should also announce the rate of women among their members, their board and committees and adopt policies to reach gender balance and use quotas as well.

We think that a domino effect would follow and other measurable actions in favor of gender equity would emerge.

The improvement of numbers and balanced ratios could enable more profound changes, which would improve women doctors’ sense of belonging and inclusion:

- Work conditions to allow personal and professional women fulfillment would change. Women might have specific needs including those related to maternity. Those measures include “breast-feeding or mother room” for women who breast-feed, childcare facilities for doctors and access to part time work. The “breast-feeding room” should be the norm in any University, Scientific Conference and learned scientific society. We also believe that allowing men to commit fully in their parenthood (especially through paternity leave extension) would improve quality of life at work for both genders and reduce discriminations related to maternity.
- Specific classes and courses about unconscious gender biases should be part of the youngest doctors’ curriculum.

We are aware that the gender gap is rooted in profound and societal dynamics that need to be modified too. Educational differences between males and females happen at the youngest age [10] and strong and sustainable changes are required for a durable success of what the critical care community yearns for.

From now on, research to define the most effective measures and possible obstacles to their implementation and their efficacy is warranted.

We know that our intensive care community is highly responsive and aware of what is at stake here: the improvement of equity and gender diversity will make our specialty fairer and more attractive for future generations.

## Data Availability

Not applicable.
